# Unique Insights into How Plants and Soil Microbiomes Interact Are at Our Fingertips

**DOI:** 10.1128/msystems.00589-22

**Published:** 2022-08-17

**Authors:** Jean Armengaud

**Affiliations:** a Université Paris-Saclay, CEA, INRAE, Département Médicaments et Technologies pour la Santé (DMTS), SPI, Bagnols-sur-Cèze, France

**Keywords:** agriculture, interactions, mass spectrometry, metaproteomics, metaexoproteomics, microbial communities, microbiome, plant, rhizosphere, symbiosis, plant-microbe interactions, protein function, rhizosphere-inhabiting microbes

## Abstract

Global warming endangers our world, with probably a drastic drop in food production as one of the first vital consequences for humanity. To maintain or improve quality and sustainable yields, the burning imperative for agriculture is to rapidly integrate the essential component for plant development and soil regeneration, namely, the soil microbiome. Although enormous progress has been made in identifying the components of this microbiome, the way in which they interact with each other and with plants remains poorly understood. Lidbury I, Raguideau S, Borsetto C, Murphy A, et al. (mSystems 7:e00025-22, 2022, https://doi.org/10.1128/mSystems.00025-22) illustrate how metaproteomics helps define key interactions between plants and microorganisms at the rhizospheric interface. The many extracellular proteins identified and quantified by this methodology uniquely explain the observed phenotype. This study shows that the adoption of metaproteomics is no longer an option that microbiologists should consider but a must!

## COMMENTARY

Microbial communities can be defined as assemblages of cooccurring and potentially interacting microorganisms present in a given habitat. Indeed, since the beginning of the existence of life, living organisms have been always strongly interconnected. In other words, life is not just an inventory of organisms but above all an incredible mass of interactions. Therefore, microbial communities can only be fully understood when analyzing the community as a whole with particular emphasis on the interconnections. Among the different types of relationships between organisms, mutualism describes the ecological interaction between two or more species where each species has a net benefit. The plant-soil interface that is influenced by root secretions, the rhizosphere, is a hot spot for mutualistic interactions between plant and microorganisms (1). In some cases, plant-specific root nodules host symbiotic microorganisms as is the case for *Frankia* and actinorhizal plants. However, in most cases, interactions with free-living microorganisms are much more elusive and difficult to assess while being crucial (2).

Microorganisms have shaped our planet and have colonized a wide variety of habitats. They play a crucial role in the biogeochemical cycles of elements such as carbon and nitrogen and are essential for soil fertility and plant health (3). For this reason, understanding soil microbial communities is of utmost interest in the context of protecting the environment, especially forests that buffer climate change, and improving agriculture. Better consideration of this component should improve crop quality and yields, without compromising sustainability (4). Metataxonomics based on 16S rRNA gene amplicon sequencing and shotgun metagenomics have been successfully applied to soil and plant rhizosphere, resulting in a large and rich body of information about the organisms present (5). However, the vogue for omics technologies based on nucleic acid sequencing over the past 2 decades has blinded the scientific community that has focused on assessing the functional potential encoded in genomes. The molecular explanation of the phenotype or its modeling is necessarily imprecise if the information is too fuzzy or is only a theoretical, bland simulacrum. The identification of the molecules explaining the phenotype can only be partially obtained by metabolomics, and unfortunately, the identified metabolites cannot be traced back to the microorganisms that produce them. As a result, only an average metabolic view is obtained and is currently far from comprehensive enough. Metaproteomics, which focuses on the identification and quantification of proteins present in a complex sample, seems to be the odd-one-out among omics technologies, but it is crucial (6). Indeed, as it focuses on the proteins that are the real workhorses of the cells, metaproteomics makes the genuine link between the potential encoded by the genome and the observed phenotype shaped by the proteins (7). This methodology is based on the identification and quantification of a myriad of short segments of protein sequences by high-resolution tandem mass spectrometry (MS/MS). Among those sequences, taxon-specific peptides make it possible to identify the taxa present in the sample. The data set can be leveraged to estimate the ratio of identified taxa in terms of biomass (8). Moreover, the peptides hold functional information that can be traced back to the organism that produced the corresponding proteins, giving rise to a precise molecular description of the phenotype of at least the most abundant taxa. Interestingly, metaproteomics can be advantageously applied to the exoproteome of any biological system. The exoproteome corresponds to the extracellular proteins, i.e., found outside the cells, either secreted or resulting from cell lysis (9). Studying this specific compartment can generate significant insights into the mechanisms deployed by microorganisms to compete for growth-limiting nutrients and interact with their environment.

Lidbury and collaborators (10) applied metaproteomics on exoproteomes to identify the most active microbial taxa in the rhizosphere of the oilseed rape and understand the main metabolic interactions at work. They reported an improved protocol for extracting extracellular proteins from rhizosphere soil. Once extracted, the proteins were subjected to denaturing electrophoresis and trypsin proteolysis. The resulting peptides were characterized by high-resolution tandem mass spectrometry. The attribution of the peptide sequences to the recorded MS/MS spectra was performed by a two-round search, a strategy that has already proven effective for this type of immensely complex samples (11): a first search was done against a database built with metagenome data acquired on the same soil sample; a second search was then carried out against a smaller database containing only the most probable protein sequences. The authors established the relevance of their method with a preliminary experiment under laboratory-controlled conditions consisting of the growth of *Brassica rapa* in a sand:soil mixture inoculated with a Pseudomonas putida strain. They proved that the exoproteome of the bacterium differs when in contact with the plant compared to when there is no plant. As assessed by metaproteomics, the active microbial community structure in the *Brassicaceae* rhizosphere of field-grown oilseed rape contrasts with that of the surrounding bulk soil. The same is observed at the functional level assessed by the protein activities. Specifically, they documented with this field sampling that autochthonous *Pseudomonadaceae* are highly active in the rhizosphere. Excreted ABC-transporter binding proteins and hydrolytic enzymes reflect the type of metabolism of rhizosphere-associated microorganisms. Several proteins serving as markers of phosphate depletion in soil were overdetected, highlighting the direct link of metaproteomics with the key parameter that drives this biological system.

The authors epitomized the difficulties that microbiologists face in identifying interactions when relying solely on metagenomic data. On the other hand, metaproteomics is easily applicable to assess the spatiotemporal dynamics of microbial communities in terms of structure and function. Obviously, there is still a lot of room to further improve this methodology. The current efforts to collectively advance the quantification of taxa of the three domains of life, their proteins, their pathways, and their biological functionalities are noteworthy (12). Ultimately, because of its unique contribution to understanding the functioning of complex microbial systems, microbiologists should embrace metaproteomics methodology to gain valuable insights into their favorite research topics and improve microbiome engineering.
